# Carcass traits, meat yield and fatty acid composition of adipose tissues and *Supraspinatus* muscle in goats fed blend of canola oil and palm oil

**DOI:** 10.1186/s40781-015-0076-y

**Published:** 2015-12-07

**Authors:** K. D. Adeyemi, M. Ebrahimi, A. A. Samsudin, A. B. Sabow, A. Q. Sazili

**Affiliations:** Department of Animal Science, Faculty of Agriculture, Universiti Putra Malaysia, UPM Serdang, 43400 Selangor Malaysia; Halal Products Research Institute, Universiti Putra Malaysia, 43400 UPM Serdang, Selangor Malaysia; Laboratory of Animal Production, Institute of Tropical Agriculture, Universiti Putra Malaysia, 43400 UPM Serdang, Selangor Malaysia; Department of Veterinary Preclinical Sciences, Faculty of Veterinary Medicine, Universiti Putra Malaysia, 43400 UPM Serdang, Selangor, Malaysia; Department of Animal Production, University of Ilorin, PMB 1515, Ilorin, Nigeria; Department of Animal Resource, University of Salahaddin, Erbil, Kurdistan Region Iraq

**Keywords:** Fat color, Mesentery, Omental, Perirenal, Subcutaneous

## Abstract

**Background:**

Dietary fats can alter the deposition and distribution of body fats in ruminants. The deposition and distribution of body fat play a vital role in the quality of ruminant carcasses and are of great commercial value since they influence the profitability and consumer acceptability of ruminant meat. The current study examined the effects of dietary blend of 80 % canola oil and 20 % palm oil (BCPO) on carcass characteristics, meat yield and accretion of fatty acid (FA) in subcutaneous, omental, perirenal, and mesentery adipose depots and *m. supraspinatus* (SS) in goats.

**Methods:**

Twenty four Boer crossbred bucks (BW 20.54 ± 0.47 kg) were randomly assigned to diets containing on DM basis 0, 4 and 8 % BCPO, fed for 100 d and harvested.

**Results:**

Diet had no effect (*P >* 0.05) on slaughter weight, dressing percentage, carcass and non-carcass components, meat yield, color, moisture and carotenoid contents and weight of adipose tissues in goats. The proportion of C18:1n-9 and *cis-*9 *trans*-11 CLA in the omental, perirenal and SS was higher (*P <* 0.05) in goats fed 4 and 8 % BCPO compared with the control goats. Dietary BCPO reduced (*P* < 0.05) the proportion of C14:0 in the omental, perirenal and mesentery depots, C18:0 in the perirenal depot, C16:0 in the SS and C16:1n-7 in the SS, omental and perirenal tissues. Dietary BCPO enhanced the proportion of C18:1 *trans*-11 Vaccenic and C18:3n-3 in SS and C20:5n-3 in SS and mesentery depot. No significant changes were found in the FA composition of subcutaneous depot.

**Conclusions:**

Results indicate that dietary BCPO can be utilized to alter the FA composition of adipose tissues without detrimental effects on carcass characteristics in goats. Nonetheless, dietary BCPO is not an effective repartitioning agent for body fats in goats.

## Background

Dietary fat is a popular means of increasing the energy density of diet in ruminant nutrition and can influence adipose depots and carcass composition [1–3]. Dietary fat can also be used to modify the fatty acid composition of meat and milk to fulfill the nutritional demands of consumers [4, 5]. The deposition and distribution of fat play a vital role in the quality of ruminant carcasses [2, 3, 6] and are of great commercial value since they influence profitability and consumer acceptability of ruminant meat [3, 6].

Goats deposit more visceral fat and less subcutaneous, inter and intra muscular fat compared with sheep and cattle [7, 8]. The deposition of more visceral fats aside its effect on product quality is expensive and represent a waste of dietary energy [3, 7, 8]. In addition, a poor subcutaneous fat cover decreases grading of goat carcasses and could instigate carcass evaporative losses [7, 8]. Albeit, the low intramuscular fat in goat meat is concordant with the present day consumers’ demands [7], a low intramuscular fat is responsible for the low juiciness and tenderness of goat meat [8, 9].

Although, there are large discrepancies, dietary fat can influence deposition of body fats and fatty acid composition of adipose tissue in ruminants [2, 3, 10]. In this respect, conjugated linoleic acid (CLA) has been identified as a potent modulator and repartitioning agent in fat metabolism [11, 12]. The CLA can be synthesized in the rumen by the biohydrogenation of linoleic and linolenic acids [13, 14]. The CLA can also be synthesized endogenously in the tissue by the action of Δ-9 desaturase on *trans*-vaccenic acid [13, 14]. The C18:1 *trans*-11 Vaccenic is a mutual intermediate product of biohydrogenation of C18:1n-9, C18:2n-6 and C18:3n-3 [4, 13, 14]. Thus, oils rich in linoleic, linolenic and/or oleic acids could alter adipose depots in ruminants [4, 13]. Canola oil is an excellent source of oleic (59 %), linoleic (21 %) and linolenic (11 %) [15]. Palm oil contains about 40 % oleic acid and 11 % linoleic acid [16]. Given the attributes of both canola and palm oils, it will be of great significance for goat meat production to examine if the blend of palm oil and canola oil could affect lipid metabolism and partitioning of body fat in goats. The efficacies of various combinations of canola oil and palm oil on *in vitro* rumen fermentation have been investigated [Adeyemi et al. unpublished] and it has been shown that the blend of 80 % canola oil and 20 % palm oil (BCPO) had no detrimental effect on *in vitro* [17] and *in vivo* rumen fermentation and growth performance in goats [18] and yielded substantial ruminal biohydrogenation intermediates. Nonetheless, we are unaware of any study that has examined the effect of blend of canola oil and palm oil on carcass traits and tissue fatty acids in goats. Thus, the objective of this study was to determine the effects of graded levels of blend of 80 % canola oil and 20 % palm oil on fat deposition, carcass traits, tissue composition of primal cuts, fat characteristics and the fatty acid composition of omental, subcutaneous, perirenal and mesentery adipose tissues and *supraspinatus* muscle in goats.

## Methods

### Animal welfare

The study was conducted following the guidelines of research policy of the Universiti Putra Malaysia on animal welfare and ethics.

### Experimental animals and diet

Twenty four Boer bucks (4–5 months old, 20.54 ± 0.47 kg body weight) were used in the study. Each animal was individually housed in wooden slatted floor pens (1.20 m × 0.80 m × 0.70 m) equipped with feeding and drinking facilities. The animals were randomly assigned to dietary treatments containing on dry matter basis 0, 4 or 8 % BCPO and fed for 100 d following two weeks of adaptation. All diets were formulated to meet the nutritional requirements of growing goats based on NRC [19] recommendations and animals were fed twice daily with ad libitum access to clean water. The proximate composition of the diets was determined following the method of AOAC [20] while acid detergent fibre (ADF) and neutral detergent fibre (NDF) were determined following the method of Van Soest et al. [21]. The ingredients and chemical composition of dietary treatments are presented in Table 1 while the fatty acid (FA) composition of the diets is presented in Table 2.

**Table 1:** Ingredients and chemical composition of dietary treatments

Ingredient (% DM)	Level of BCPO^a^ (%)		
	0	4	8
Fresh OPF^b^	40.00	40.00	40.00
OPF^b^ silage	10.00	10.00	10.00
Corn grain	22.00	12.50	6.00
Soybean meal	17.00	19.00	20.00
Rice bran	2.00	7.00	10.50
Palm kernel cake	7.50	6.00	4.00
BCPO^a^	0.00	4.00	8.00
Limestone	0.50	0.50	0.50
Salt	0.50	0.50	0.50
Mineral-vitamin premix^c^	0.50	0.50	0.50
Chemical composition, % DM			
Dry matter	67.70	67.90	68.07
Organic matter	93.16	93.42	93.55
Metabolizable energy, MJ/Kg DM^b^	11.59	11.61	11.62
Crude Protein,	14.27	14.37	14.39
Ether extract	3.06	6.56	11.45
Crude fibre	28.48	27.64	26.81
ADF	35.04	33.28	32.52
NDF	63.52	62.67	62.06
Nitrogen free extract	16.56	13.97	12.45
Ca	1.02	1.05	1.04
P	0.52	0.54	0.54
Total carotenoid (mg/kg)	14.81	16.71	19.86

^a^blend of 80 % canola oil and 20 % palm oil. ^b^Oil palm frond. ^c^Contained (g/kg) CuSO_4_.5H_2_O, 70; ZnSO_4_.7H_2_O, 240; FeSO_4_.7H_2_O, 170; MnSO_4_.5H_2_O, 290; (mg/kg) CoCl_2_.6H_2_O 510; KI, 220; NaSeO, 130; vitamin B_1_, 450; pantothenic acid, 750; vitamin K3, 150; folic acid, 15; vitamin B_12_, 0.9; vitamin B_5_, 1,050; (IU) vitamin D_3_, 324,000; vitamin A, 620,000. ^b^calculated

**Table 2:** Fatty acid composition (g/kg DM) of dietary treatments

	Level BCPO^a^ (%)		
Parameter	0	4	8
C12:0, lauric	0.01	0.03	0.04
C14:0, myristic	0.53	0.51	0.51
C16:0, palmitic	2.79	5.98	7.78
C16:1, palmitoleic	0.08	0.11	0.15
C18:0, stearic	0.56	1.12	1.43
C18:1n-9, oleic	3.82	14.87	25.23
C18:2n-6, linoleic	7.05	11.87	13.07
C18:3n-3, linolenic	1.06	2.61	4.23
Total FA	15.89	37.09	52.27
FA sums and ratios			
∑SFA	3.89	7.79	9.79
∑UFA	12.00	29.31	42.52
∑PUFA	8.11	14.49	16.19
n-6:n-3	6.65	4.54	3.08

^a^blend of 80 % canola oil and 20 % palm oil. ∑SFA = (C12:0 + C14:0 + C16:0 + C18:0), ∑UFA = (C16:1+ C18:1 + C18:2n-6 + C18:3n-3), n-6:n-3 = (C18:2n-6÷C18:3n-3)

### Slaughtering procedure and carcass analysis

The animals were fasted for 12 h with free access to water, weighed and slaughtered in a commercial abattoir according to the Halal procedure as outlined in MS1500:2009 [22]. After slaughter, the head, fore- and hind limbs were removed at the atlanto-occipital, carpal and tarsal joints respectively. The animals were skinned while suspended by their achilles tendon and later eviscerated. Carcass and non-carcass components were weighed. Subcutaneous fat was sampled from inguinal region, omental fat was sampled near its free part close to the rumen (*omentum majus*), perirenal fat was sampled near the kidney on its great curve (between the renal fascia and renal capsule), and mesentery fat was sampled between the ileum and jejunum while pelvic fat was sampled at the left pelvic region. The *Supraprinatus* muscle was sampled from the right forelimb. The weight of gut fill was estimated by subtracting the weight of empty gut from the weight of full gut. Empty body weight was estimated as the difference between the slaughter weight and weight of gut fill. Dressing percentage (based on full live body weight and empty body weight) was estimated as described by Kadim et al. [23]. Dressed carcasses were weighed (hot carcass weight) within 45 min postmortem, chilled at 4 °C for 24 h and reweighed (cold carcass weight). Chilling loss was estimated as the difference between hot and cold carcass weight expressed as percentage. Carcasses were split along the midline into right and left halves. The right half was separated into neck, shoulder, breast flank, loin and leg cuts. Each cut was separated into lean, fat and bone. *Longissimus* muscle area (rib eye area) was measured between the 12th and 13th ribs using tracing paper and estimated using two-dimension polygons area calculator software as described by Branscome and Jesseman [24]. Carcass linear measurements were determined as outlined by Fisher and De Boer [25] and Laville et al. [26]. These measurements were used to estimate carcass compactness (cold carcass weight/internal carcass length), shoulder compactness (shoulder weight/length of shoulder) and leg compactness (leg weight/leg length) as described by Carrasco et al. [27]. Saleable meat yield (SMY) was estimated as SMY% = (100 – fat %) × (meat:bone)/ ((meat:bone) + 1) as outlined by Purchas [28].

### Fat color and moisture content

The color coordinates of adipose tissues were measured with a ColorFlex spectrophotometer (Hunter Lab Reston, VA, USA) based on the International Commission on Illumination (CIE) Lab-values (also known as L*, a*, b*) with D65 illuminant and 10° standard observer. The device was calibrated against black and white reference tiles prior to use. The moisture content of the adipose tissue was measured in accordance to the method of AOAC [20].

### Total carotenoid

Total carotenoid in feed and adipose tissues was determined in accordance to the method described by Okonkwo [29]. One gram of each sample was homogenized with 5 mL acetone. The content was stirred for 30 min and two 2.5 mL aliquot of acetone was used to rinse the flask and re-extract the residue. The extracts were pooled and 1 mL of deionized water was added. The mixture was transferred into 5 mL n-hexane and centrifuged at 3000 g for 10 min. The absorbance of the hexane layer was read at 450 nm using spectrophotometer (Secomam, Domont, France). Total carotenoid content was estimated by the following formula

$$ \mathrm{Conc}.\left(\upmu \mathrm{g}/\mathrm{g}\right)=\left(\mathrm{A} \times \mathrm{V} \times 1{0}^4\right)/\left(2592 \times \mathrm{W}\right) $$

$$ \mathrm{Where}\ \mathrm{A}=\mathrm{absorbance} $$

$$ \mathrm{V}=\mathrm{Volume}\ \mathrm{of}\ \mathrm{n}-\mathrm{hexane}\left(\mathrm{mL}\right) $$

$$ \mathrm{W}=\mathrm{Weight}\ \mathrm{of}\ \mathrm{sample} $$

### Fatty acid (FA) analysis

The total lipids in feed and tissue samples was extracted in chloroform:methanol (2:1, v/v) mixture following the method of Folch et al. [30] modified by Rajion et al. [31]. The fatty acids were transmethylated into their fatty acid methyl esters (FAME) using 0.66 N KOH in methanol and 14 % methanolic boron trifluoride (BF_3_) in accordance with the method of AOAC [20]. Heneicosanoic acid was used as the internal standard. The FAME was separated in a gas chromatograph (Agilent 7890A) equipped with a flame ionization detector (FID) and a splitless injector. The column used was fused silica capillary (Supelco SP-2560, 100 m, 0.25 mm ID, 0.20 mm film thickness). High purity nitrogen was used as the carrier gas at 40 mL/min. Compressed air and high purity hydrogen were used for the FID in the chromatograph. To facilitate optimal separation, the oven temperature was set at 100 °C, for 2 min and warmed to 170 °C at 10 °C/min, held for 2 min, warmed to 230 °C at 5 °C/min, and then held for 20 min. Identification of sample fatty acids was done by comparing the relative retention times of FAME peaks from samples with those of standards. The indices of Δ 9 desaturase enzyme activities (ID16 and ID18) and elongase enzyme activity in tissues were estimated as described by Malau-Aduli et al. [32] as follows: ID16 = 100(C16:1)/(C16:0 + C16:1), ID18 = 100(C18:1)/C18:0 + C18:1) and Elongation index = 100[(C18:0 + C18:1 n-9)/(C18:0 + C18:1n-9 + C16:0 + C16:1)].

### Statistical analysis

The experiment followed a completely randomized design. Data obtained for all parameters were subjected to the generalized linear model (GLM) procedure of SAS [33]. Slaughter weight was used as a covariate. However, the covariate analysis was not significant. Orthogonal contrasts (linear and quadratic effects) were tested with coefficients estimated based on the level of dietary oil.

## Results

### Ingredients, chemical and fatty acid composition of experimental diets

The ingredients and chemical composition of the experimental diets are shown in Table 1 while the fatty acid composition is shown in Table 2. In each treatment, oil palm fronds (OPF) accounted for 50 % of the total ration. The OPF is obtained from oil palm (*Elaeis guinensis*) tree and constitute one of the main by-products of the oil palm industry in Malaysia [34]. The OPF is made up of three main components namely the petioles, leaflets and rachis. The petiole is the main structure of the OPF and accounts for about 70 % of the dry matter (DM) of OPF. The proximate analysis of OPF used in the current study showed that it contained on DM basis: 4.6 % crude protein, 2 % ether extract, 39 % crude fiber, 78.5 % NDF, 56.4 % ADF, 3.2 % ash, and 5.7 MJ/kg metabolizable energy. As a percentage of total FA, the OPF contained 31.3 % C16:0, 5.4 % C16:1, 4.4 % C18:0, 20.9 % C18:1n-9, 16 % C18:2n-6, 17 % C18:3n-3 and 5 % other FA. The concentrate portion of the diets consisted of various ingredients which were adjusted to make the experimental diets isocaloric and isonitrogenous since the addition of graded level of BCPO would increase the energy content of the diet. This was done to prevent confounding experimental results that could accompany changing the protein and energy status of the diets. Thus, responses by animals can be attributed to the addition of BCPO to the diets. In addition, the varied ingredients (corn, soybean meal, palm kernel cake and rice bran) are not fat sources and thus contributed 1.74 and 1.67 % FA to the total FA in the 4 and 8 % BCPO diets respectively. Hence, the FA composition of such ingredients would not be a confounding factor in the current trial. Similar experimental design (control diet vs. oil based diets) which necessitates altering the composition of other dietary ingredients was used in goats [35], sheep [36, 37] and cattle [1, 2, 5, 38, 39]. The chemical composition of the experimental diets met the minimum requirements for growing goats recommended by NRC [19]. As expected, the total FA in the supplemented diets was greater than the control diet. Notably, addition of BCPO increased the concentration (g/kg DM) of C16:0, C18:1n-9, C18:3n-3 and C18:2n-6 but lowered the n6/n3 ratio.

### Carcass traits and fat depots

The carcass traits of goats fed varying levels of BCPO are presented in Table 3. Dietary BCPO did not affect (*P >* 0.05) live, empty body and carcass weights, dressing percentage and meat yield in goats. Similarly, the rib eye area and other carcass linear measurements (Table 3), non-carcass components (Table 4) and proportion, weight and tissue composition of primal cuts (Table 5) were unaffected (*P >* 0.05). The back fat thickness, intramuscular fat (Table 3) of *supraspinatus* muscle, and the weights of perirenal, omental, mesentery and pelvic adipose depots (Table 4) were not influenced (*P >* 0.05) by diets. The carotenoid and moisture contents and color coordinates of all adipose tissues (Table 6) were similar (*P >* 0.05) across dietary treatments.

**Table 3:** Least square means for the carcass traits of goats fed graded levels of blend of 80 % canola oil and 20 % palm oil

	Level of BCPO^a^ (%)				*P* value		
Parameter	0	4	8	SEM	Overall	Linear	Quadratic
Slaughter weight (Kg)	29.58	32.19	31.33	0.91	0.144	0.064	0.511
Empty body weight (Kg)	24.87	26.18	25.72	0.81	0.515	0.284	0.691
Hot carcass weight (kg)	13.56	14.21	13.78	0.42	0.542	0.408	0.467
Cold carcass weight (kg)	13.21	13.90	13.48	0.41	0.497	0.352	0.469
Chilling loss (%)	2.60	2.17	2.19	0.21	0.304	0.127	0.955
Dressing percentage^b^	45.81	44.41	44.02	1.62	0.366	0.177	0.694
Dressing percentage^c^	54.56	54.52	53.55	0.94	0.701	0.654	0.479
Back fat thickness (mm)	2.17	2.28	2.29	0.20	0.844	0.598	0.848
Rib eye area (cm^2^)	13.08	13.92	13.41	0.54	0.568	0.407	0.527
Saleable meat yield (%)	68.86	69.24	69.34	1.45	0.700	0.581	0.620
IMF^d^	2.78	2.76	2.79	0.03	0.221	0.551	0.761
*Linear measurements*							
Carcass length (cm)	79.34	78.00	78.40	1.09	0.691	0.428	0.805
Internal carcass length (cm)	71.34	70.00	70.40	1.09	0.691	0.428	0.805
Carcass compactness (g/cm)	184.78	200.56	192.14	8.62	0.477	0.315	0.515
Shoulder width (cm)	45.33	46.33	45.67	0.94	0.757	0.584	0.634
Shoulder length (cm)	24.33	23.17	22.84	0.92	0.403	0.415	0.581
Shoulder compactness (g/cm)	177.97	189.47	190.89	7.20	0.617	0.569	0.511
Leg width (cm)	40.00	40.23	40.53	0.49	0.754	0.548	0.681
Leg length (cm)	46.03	46.41	46.00	0.92	0.457	0.415	0.581
Leg compactness (g/cm)	95.22	98.84	92.47	7.47	0.837	0.963	0.568
*Carcass composition*							
Lean (%)	68.60	69.41	69.08	0.86	0.453	0.366	0.391
Bone (%)	23.81	22.93	22.56	0.42	0.132	0.620	0.057
Subcutaneous fat (%)	2.33	2.54	2.58	0.21	0.687	0.597	0.511
Intermuscular fat (%)	4.91	5.34	5.44	0.44	0.686	0.589	0.515

^a^blend of 80 % canola oil and 20 % palm oil. ^b^estimated based on full live body weight. ^c^estimated based on empty body weight (Kadim et al., 2003). ^d^intramuscular fat of *supraspinatus* muscle. SEM = standard error of means. Values are least square means for 8 goats per diet

**Table 4:** Least square means for the weight of non-carcass components from goats fed graded levels of blend of 80 % canola oil and 20 % palm oil

	Level of BCPO^a^ (%)				*P* value		
Parameter (Kg)	0	4	8	SEM	Overall	Linear	Quadratic
Head	2.36	2.46	2.25	0.09	0.257	0.9545	0.103
Feet	1.00	1.04	0.98	0.03	0.379	0.871	0.171
Skin	3.40	3.65	3.23	0.15	0.168	0.818	0.063
Heart	0.11	0.12	0.12	0.01	0.785	0.494	0.936
Lungs	0.24	0.22	0.23	0.01	0.606	0.357	0.710
Kidney	0.07	0.07	0.08	0.01	0.548	0.325	0.463
Liver	0.43	0.43	0.45	0.02	0.612	0.830	0.338
Reticulo-rumen	1.25	1.29	1.46	0.07	0.120	0.179	0.118
Intestine	0.84	0.87	1.01	0.05	0.187	0.176	0.113
Omental fat	0.24	0.29	0.27	0.02	0.357	0.188	0.583
Perirenal fat (K)	0.10	0.12	0.09	0.02	0.654	0.763	0.389
Mesentery fat	0.24	0.26	0.24	0.01	0.195	0.318	0.129
Pelvic fat (P)	0.15	0.19	0.18	0.03	0.440	0.243	0.348
Heart fat (H)	0.07	0.07	0.07	0.01	0.771	0.825	0.810
KPH%^b^	2.42	2.73	2.52	0.12	0.423	0.560	0.240

^a^blend of 80 % canola oil and 20 % palm oil. ^b^estimated based on percentage of cold carcass weight. SEM = standard error of means. Values are least square means for 8 goats per diet

**Table 5:** Least square means for the proportion, weight and tissue composition of primal cuts from goats fed graded levels of blend of 80 % canola oil and 20 % palm oil

	Level of BCPO^a^ (%)				*P* value		
Parameters	0	4	8	SEM	Overall	Linear	Quadratic
Proportion (%)^b^							
Neck	6.43	6.54	6.75	0.52	0.5722	0.909	0.751
Shoulder	32.77	31.58	31.53	2.54	0.8369	0.656	0.881
Loin	10.06	9.78	10.16	0.28	0.9263	0.643	0.335
Breast	17.56	19.35	19.21	0.57	0.8413	0.987	0.817
Leg	33.16	32.73	32.34	2.61	0.9917	0.747	0.822
Shoulder (kg)	4.33	4.39	4.25	0.29	0.6252	1.000	0.901
Lean (%)	71.73	73.17	73.33	4.65	0.3524	0.234	0.981
Bone (%)	22.18	21.71	21.58	2.18	0.6556	0.242	0.968
Fat (%)	5.81	5.12	5.08	0.97	0.7557	0.569	0.981
Loin (Kg)	1.33	1.36	1.37	0.07	0.2934	0.715	0.927
Lean (%)	67.40	67.97	67.89	2.69	0.8436	0.474	0.343
Bone (%)	21.93	20.96	20.34	0.66	0.7529	0.162	0.531
Fat (%)	10.67	11.07	11.77	2.20	0.4219	0.659	0.329
Neck (Kg)	0.85	0.91	0.91	0.04	0.5459	0.310	1.000
Lean (%)	74.72	78.20	77.22	2.52	0.3116	0.369	0.791
Bone (%)	21.64	20.18	17.27	0.99	0.3683	0.054	0.084
Fat (%)	5.55	5.17	5.52	0.48	0.6094	0.740	0.626
Leg (kg)	4.38	4.55	4.36	0.11	0.2992	0.963	0.568
Lean (%)	70.41	68.97	69.23	0.62	0.7893	0.567	0.153
Bone (%)	23.03	24.90	24.31	0.95	0.7279	0.776	0.211
Fat (%)	6.56	6.13	6.02	0.36	0.6233	0.476	0.427
Breast (kg)	2.32	2.69	2.59	0.08	0.4845	0.589	0.867
Lean (%)	62.09	61.97	62.00	2.05	0.4640	0.821	0.891
Bone (%)	29.23	30.09	29.86	0.54	0.4768	0.744	0.923
Fat (%)	8.68	7.94	8.14	2.10	0.8383	0.723	0.562

^a^blend of 80 % canola oil and 20 % palm oil. ^b^estimated based on percentage of cold carcass weight. SEM = standard error of means. Values are least square means for 8 goats per diet

**Table 6:** Least square means for color coordinates, carotenoid and moisture contents of adipose tissues from goats fed graded levels of blend of 80 % canola oil and 20 % palm oil

		Level of BCPO^a^ (%)				*P* value		
Adipose tissue	Color coordinates	0	4	8	SEM	Overall	Linear	Quadratic
Subcutaneous	L*	78.07	80.02	81.29	2.07	0.563	0.335	0.674
	a*	0.92	0.26	0.40	0.05	0.646	0.373	0.847
	b*	10.97	9.53	10.16	0.84	0.506	0.304	0.606
Omental	L*	78.14	78.71	81.03	1.17	0.235	0.226	0.199
	a*	1.04	0.96	1.00	0.25	0.318	0.384	0.239
	b*	11.01	11.03	10.57	0.38	0.660	0.632	0.423
Perirenal	L*	79.07	80.07	82.18	1.61	0.080	0.053	0.380
	a*	3.12	2.34	2.62	0.55	0.805	0.064	0.347
	b*	12.87	12.28	10.32	0.83	0.068	0.247	0.055
Mesentery	L*	80.57	79.63	77.62	1.58	0.104	0.058	0.102
	a*	1.62	1.46	1.45	0.39	0.203	0.726	0.986
	b*	11.28	13.99	11.87	0.78	0.100	0.092	0.080
Total carotenoid (mg/kg)								
Subcutaneous		0.09	0.11	0.12	0.01	0.210	0.100	0.213
Omental		0.17	0.19	0.22	0.01	0.215	0.200	0.341
Perirenal		0.19	0.24	0.26	0.01	0.223	0.180	0.119
Mesentery		0.18	0.22	0.24	0.01	0.190	0.200	0.401
Moisture content (%)								
Subcutaneous		14.87	13.45	8.04	2.63	0.233	0.248	0.196
Omental		19.04	16.66	14.56	2.08	0.374	0.956	0.178
Perirenal		18.75	13.94	11.15	2.84	0.239	0.124	0.512
Mesentery		22.68	17.04	16.92	2.81	0.324	0.149	0.977

^a^blend of 80 % canola oil and 20 % palm oil. L*, lightness, a*, redness, b*, yellowness. SEM = standard error of means. Values are least square means for 8 goats per diet

### Fatty acid composition of *Supraspinatus* muscle

The FA composition of *Supraspinatus* (SS) muscle from goats fed varying level of BCPO is shown in Table 7. Regardless of the diet, the most abundant FA in SS was C18:1n-9 followed by C16:0 and C18:0 in that order. The proportion of C16:0 and C16:1n-7 decreased (*P* < 0.05) while that of C18:1n-9, C18:1 *trans*-11, CLA cis-9 *trans*-11, C18:3n-3 and C20:5n-3n increased (*P* < 0.05) as the level of BCPO increased in diet. The proportion of C18:0, C18:2n-6 and C20:4n-6 was not influenced (*P >* 0.05) by diet. The total saturated fatty acids (SFA), the n-6/n-3 ratio and ID 16 decreased (*P* < 0.05) while the total monounsaturated fatty acids (MUFA), PUFA/SFA ratio, ID 18 and elongation index increased (*P* < 0.05) with increase in the level of BCPO in diet.

**Table 7:** Least square means for the fatty acid composition (% of total FA) of *supraspinatus* muscle in goats fed graded levels of blend of 80 % canola oil and 20 % palm oil

	Level of BCPO^d^ (%)				*P* value		
Parameter	0	4	8	SEM	Overall	Linear	Quadratic
C14:0, myristic	2.98	2.17	2.11	0.03	0.213	0.421	0.212
C16:0, palmitic	24.53^a^	21.63^b^	17.34^c^	2.14	0.011	0.031	0.330
C16:1n-7, palmitoleic	3.29^a^	2.16^b^	1.09^c^	0.01	0.046	0.002	0.175
C18:0, stearic	17.93	17.35	15.39	1.54	0.541	0.472	0.112
C18:1n-9, oleic	28.13^c^	31.93^b^	36.44^a^	3.00	0.021	0.041	0.331
C18:1 *trans*-11 Vaccenic	2.02^a^	3.02^b^	4.78^c^	0.20	0.012	0.031	0.221
CLA *cis*-9 *trans*-11	0.66^c^	1.08^b^	1.14^c^	0.02	0.012	0.012	0.221
CLA *trans*-10* cis*-12	0.68	0.76	0.79	0.22	0.556	0.213	0.451
C18:2n-6, linoleic	10.61	10.26	10.60	1.20	0.150	0.187	0.156
C18:3n-3, linolenic	0.72^a^	1.00^b^	1.45^c^	0.02	0.013	0.048	0.189
C20:4n-6, arachidonic	5.55	5.34	5.24	0.56	0.212	0.568	0.118
C20:5n-3, eicosapentaenoic	0.86^a^	1.28^b^	1.54^c^	0.02	0.045	0.042	0.091
C22:5n-3, docosapentaenoic	0.99	1.00	0.74	0.10	0.541	0.219	0.871
C22:6n-3, docosahexaenoic	1.06	1.04	1.05	0.01	0.342	0.221	0.448
Total FA (mg/g of muscle)	2.11	2.34	2.18	0.03	0.112	0.574	0.378
FA ratios and sums							
∑SFA	45.44^a^	41.15^b^	34.84^c^	4.21	0.001	0.002	0.019
∑MUFA	33.26^c^	37.11^b^	42.31^a^	3.21	0.005	0.001	0.003
∑PUFA	21.13	21.76	22.55	3.11	0.213	0.445	0.318
∑n-3	3.63^c^	4.32^b^	4.78^a^	0.76	0.014	0.047	0.231
∑n-6	16.16	15.60	15.84	1.21	0.223	0.345	0.186
n-6:n-3	4.45^a^	3.61^b^	3.31^c^	0.50	0.034	0.041	0.176
UFA:SFA	1.20^a^	1.43^b^	1.86^c^	0.07	0.013	0.045	0.124
PUFA:SFA	0.46^c^	0.52^b^	0.65^a^	0.01	0.025	0.019	0.332
Desaturase and elongase indices							
ID16	11.83^a^	9.08^b^	5.91^c^	1.20	0.032	0.001	0.664
ID18	61.07^c^	64.29^b^	70.30^a^	5.11	0.041	0.045	0.716
Elongation Index (EI)	68.46^a^	67.44^a^	73.76^b^	6.72	0.045	0.043	0.212

^a,b,c^means having different superscript along the a row are significantly different (*P* < 0.05). ∑SFA = (C14:0 + C16:0 + C18:0), ∑MUFA = (C16:1+ C18:1+ C18:1 *trans*-11), ∑UFA = (C16:1+ C18:1+ C18:1 *trans*-11+ CLA *cis*-9 *trans*-11+ CLA *cis*-12 *trans*-10 + ∑n-3 + ∑n-6), ∑PUFA = (C18:1 *trans*-11+ CLA *cis*-9 *trans*-11+ CLA *cis*-12 *trans*-10 + ∑n-3 + ∑n-6), ∑n-3 = (C18:3n-3 + C20:5n-3 + C22:5n-3 + C22:6n-3), ∑n-6 = (C18:2n-6 + C20:4n-6) n-6:n-3 = (C18:2n-6 + C20:4n-6) ÷ (C18:3n-3 + C20:5n-3 + C22:5n-3 + C22:6n-3), UFA:SFA = (∑UFA)/∑SFA), PUFA:SFA = (∑PUFA/∑SFA). ID16, (Δ 9 desaturase enzyme activity on C16:0) = 100(C16:1)/(C16:0 + C16:1), ID18, (Δ 9 desaturase enzyme activity on C18:0) = 100(C18:1)/C18:0 + C18:1). EI = 100[(C18:0 + C18:1 n-9)/(C18:0 + C18:1n-9 + C16:0 + C16:1)]. SEM = standard error of means. Values are least square means for 8 goats per diet. ^d^blend of 80 % canola oil and 20 % palm oil

### Fatty acid composition of adipose tissues

The FA composition of omental, perirenal, mesentery and subcutaneous adipose tissues from goats fed graded levels of BCPO is shown in Tables 8, 9, 10and 11 respectively. The major fatty acids in all examined adipose tissues were C16:0, C18:0 and C18:1n-9.

**Table 8:** Least square means for the fatty acid (FA) composition (% of total FA) of omental adipose tissue in goats fed graded levels of blend of 80 % canola oil and 20 % palm oil

	Levels of BCPO^d^ (%)				*P *value		
Parameter	0	4	8	SEM	Overall	Linear	quadratic
C14:0, myristic	5.55^a^	4.18^b^	4.04^b^	0.30	0.003	0.001	0.757
C16:0, palmitic	26.67	21.87	24.57	1.92	0.232	0.158	0.331
C16:1n-7, palmitoleic	2.94^a^	2.25^b^	2.01^c^	0.12	<.0001	<.0001	0.176
C18:0, stearic	31.24	35.41	32.42	1.86	0.092	0.142	0.096
C18:1n-9, oleic	24.99^b^	29.67^a^	31.07^a^	2.85	0.006	0.037	0.730
C18:1 *trans*-11 Vaccenic	4.77^a^	0.78^b^	0.61^b^	0.79	0.001	0.0004	0.882
CLA *cis*-9 *trans*-11	0.96^b^	1.29^a^	1.29^a^	0.07	0.004	0.001	0.940
CLA *trans*-10 *cis*-12	0.29	0.31	0.39	0.04	0.197	0.200	0.198
C18:2n-6, linoleic	3.14	3.40	3.96	0.42	0.387	0.306	0.357
C18:3n-3, linolenic	0.17	0.25	0.18	0.02	0.055	0.189	0.039
C20:4n-6, arachidonic	0.11	0.10	0.11	0.01	0.560	0.418	0.483
C20:5n-3, eicosapentaenoic	0.08^c^	0.16^b^	0.56^c^	0.08	0.001	0.008	0.002
C22:5n-3, docosapentaenoic	0.20	0.29	0.40	0.13	0.574	0.383	0.561
C22:6n-3, docosahexaenoic	0.15	0.08	0.09	0.05	0.463	0.223	0.894
Total FA (mg/g of fat)	651.60	610.23	697.51	117.30	0.871	0.987	0.604
FA ratios and sums							
∑SFA	63.46	61.46	61.02	2.68	0.792	0.669	0.601
∑MUFA	31.45	32.69	33.69	2.83	0.959	0.887	0.806
∑PUFA	5.09^a^	5.86^b^	6.96^c^	0.51	0.018	0.048	0.145
∑n-3	0.60	0.78	1.22	0.19	0.096	0.112	0.128
∑n-6	3.24	3.49	4.07	0.42	0.397	0.324	0.350
n-6:n-3	9.85^a^	6.26^b^	3.90^c^	1.74	0.047	0.036	0.350
UFA:SFA	0.58	0.62	0.66	0.05	0.665	0.448	0.632
PUFA:SFA	0.08	0.10	0.11	0.01	0.102	0.127	0.124
Elongase and desaturase indices							
ID16	9.90	9.30	7.56	1.34	0.116	0.671	0.094
ID18	44.44	45.59	48.95	2.16	0.324	0.567	0.713
Elongation index (EI)	65.50	72.96	70.49	3.01	0.772	0.712	0.546

^a,b,c^means having different superscript along the a row are significantly different (*P* < 0.05). ∑SFA = (C14:0 + C16:0 + C18:0), ∑MUFA = (C16:1+ C18:1+ C18:1 *trans*-11), ∑UFA = (C16:1+ C18:1+ C18:1 *trans*-11+ CLA *cis*-9 *trans*-11+ CLA *cis*-12 *trans*-10 + ∑n-3 + ∑n-6), ∑PUFA = (C18:1 *trans*-11+ CLA *cis*-9 *trans*-11+ CLA *cis*-12 *trans*-10 + ∑n-3 + ∑n-6), ∑n-3 = (C18:3n-3 + C20:5n-3 + C22:5n-3 + C22:6n-3), ∑n-6 = (C18:2n-6 + C20:4n-6) n-6:n-3 = (C18:2n-6 + C20:4n-6) ÷ (C18:3n-3 + C20:5n-3 + C22:5n-3 + C22:6n-3), UFA:SFA = (∑UFA)/∑SFA), PUFA:SFA = (∑PUFA/∑SFA). ID16, (Δ 9 desaturase enzyme activity on C16:0) = 100(C16:1)/(C16:0 + C16:1), ID18, (Δ 9 desaturase enzyme activity on C18:0) = 100(C18:1)/C18:0 + C18:1). EI = 100[(C18:0 + C18:1 n-9)/(C18:0 + C18:1n-9 + C16:0 + C16:1)] SEM = standard error of means. Values are least square means for 8 goats per diet. ^d^blend of 80 % canola oil and 20 % palm oil

**Table 9:** Least square means for the fatty acid (FA) composition (% of total FA) of perirenal adipose tissue in goats fed graded levels of blend of 80 % canola oil and 20 % palm oil

	Level of BCPO^d^ (%)				*P* value		
Parameter	0	4	8	SEM	Overall	Linear	Quadratic
C14:0, myristic	6.02^a^	3.87^b^	3.85^b^	0.46	0.005	0.001	0.974
C16:0, palmitic	23.44	25.74	24.18	2.34	0.774	0.614	0.619
C16:1n-7, palmitoleic	2.94^a^	1.99^b^	1.83^c^	0.13	<.0001	<.0001	0.336
C18:0, stearic	40.18^a^	38.92^b^	30.64^c^	1.79	0.001	0.027	0.002
C18:1n-9, oleic	17.69^c^	21.03^b^	29.05^c^	1.73	0.001	0.003	0.002
C18:1 *trans*-11 Vaccenic	4.27	3.55	5.15	1.34	0.668	0.966	0.376
CLA *cis*-9 *trans*-11	0.80^b^	1.09^a^	1.11^a^	0.04	0.054	0.046	0.191
CLA *trans*-10 *cis*-12	0.36	0.36	0.33	0.15	0.578	0.369	0.603
C18:2n-6, linoleic	3.52	3.20	3.70	0.27	0.404	0.839	0.188
C18:3n-3, linolenic	0.27	0.32	0.28	0.02	0.213	0.240	0.185
C20:4n-6, arachidonic	0.08	0.06	0.08	0.12	0.702	0.757	0.440
C20:5n-3, eicosapentaenoic	0.76	0.53	0.52	0.18	0.593	0.313	0.966
C22:5n-3, docosapentaenoic	0.15	0.47	0.32	0.15	0.356	0.218	0.463
C22:6n-3, docosahexaenoic	0.11	0.30	0.24	0.16	0.705	0.751	0.445
Total FA (mg/g of fat)	544.47	673.58	692.52	124.31	0.679	0.389	0.909
FA ratios and sums							
∑SFA	69.64^a^	68.53^b^	58.67^c^	2.36	<.0001	0.004	0.0002
∑MUFA	24.91^c^	26.57^b^	36.02^a^	1.54	<.0001	0.005	0.0001
∑PUFA	5.78	6.33	6.55	0.33	0.884	0.127	0.654
∑n-3	1.29	1.63	1.36	0.29	0.709	0.517	0.613
∑n-6	3.63	3.26	3.78	0.40	0.224	0.966	0.376
n-6:n-3	2.81	2.00	2.78	0.89	0.643	0.354	0.988
UFA:SFA	0.44^c^	0.47^b^	0.71^a^	0.04	<.0001	0.005	<.0001
PUFA:SFA	0.08	0.09	0.11	0.01	0.144	0.758	0.054
Desaturase and elongase indices							
ID16	11.14^a^	7.18^b^	7.03^c^	0.90	0.020	0.031	0.123
ID18	30.57^c^	35.08^b^	48.67^a^	1.34	0.014	0.042	0.070
Elongation Index (EI)	68.68	68.37	69.64	2.19	0.859	0.706	0.901

^a,b,c^means having different superscript along the a row are significantly different (*P* < 0.05). ∑SFA = (C14:0 + C16:0 + C18:0), ∑MUFA = (C16:1+ C18:1+ C18:1 *trans*-11), ∑UFA = (C16:1+ C18:1+ C18:1 *trans*-11+ CLA *cis*-9 *trans*-11+ CLA *cis*-12 *trans*-10 + ∑n-3 + ∑n-6), ∑PUFA = (C18:1 *trans*-11+ CLA *cis*-9 *trans*-11+ CLA *cis*-12 *trans*-10 + ∑n-3 + ∑n-6), ∑n-3 = (C18:3n-3 + C20:5n-3 + C22:5n-3 + C22:6n-3), ∑n-6 = (C18:2n-6 + C20:4n-6) n-6:n-3 = (C18:2n-6 + C20:4n-6) ÷ (C18:3n-3 + C20:5n-3 + C22:5n-3 + C22:6n-3), UFA:SFA = (∑UFA/∑SFA), PUFA:SFA = (∑PUFA/∑SFA). ID16, (Δ 9 desaturase enzyme activity on C16:0) = 100(C16:1)/(C16:0 + C16:1), ID18, (Δ 9 desaturase enzyme activity on C18:0) = 100(C18:1)/C18:0 + C18:1). EI = 100[(C18:0 + C18:1 n-9)/(C18:0 + C18:1n-9 + C16:0 + C16:1)]. SEM = standard error of means. Values are least square means for 8 goats per diet. ^d^blend of 80 % canola oil and 20 % palm oil

**Table 10:** Least square means for the fatty acid (FA) composition (% of total FA) of mesentery adipose tissue in goats fed graded levels of blend of 80 % canola oil and 20 % palm oil

	Level of BCPO^d^ (%)				*P* value		
Parameter	0	4	8	SEM	Overall	Linear	Quadratic
C14:0, myristic	6.67^a^	5.70^b^	5.04^c^	0.53	0.048	0.049	0.395
C16:0, palmitic	27.06	27.05	24.22	1.18	0.170	0.334	0.104
C16:1n-7, palmitoleic	3.49	3.44	2.74	0.44	0.409	0.461	0.266
C18:0, stearic	26.10	25.28	24.42	2.56	0.898	0.814	0.693
C18:1n-9, oleic	19.97^b^	18.98^b^	26.30^a^	2.42	0.050	0.044	0.377
C18:1 *trans*-11 Vaccenic	3.48	4.27	4.87	0.73	0.422	0.569	0.239
CLA *cis*-9 *trans*-11	1.30	1.51	1.20	0.22	0.612	0.337	0.841
CLA* trans*-10* cis*-12	1.54	2.08	1.72	0.39	0.618	0.521	0.463
C18:2n-6, linoleic	3.63	3.65	3.53	0.53	0.986	0.878	0.953
C18:3n-3, linolenic	1.56	1.44	1.71	0.23	0.712	0.417	0.952
C20:4n-6, arachidonic	1.89	1.62	2.13	0.52	0.784	0.491	0.980
C20:5n-3, eicosapentaenoic	1.87^c^	2.78^b^	2.91^c^	0.39	0.043	0.011	0.051
C22:5n-3, eicosapentaenoic	1.42	1.67	2.11	0.42	0.501	0.458	0.365
C22:6n-3, docosahexanoic	1.92	1.87	2.29	0.52	0.824	0.574	0.804
Total FA (mg/g of fat)	842.23	925.87	982.87	160.09	0.827	0.804	0.577
FA sums and ratios							
∑SFA	59.83	58.03	54.09	3.24	0.402	0.353	0.328
∑MUFA	26.95	26.69	33.90	6.62	0.169	0.113	0.299
∑PUFA	15.12	16.61	17.60	1.61	0.437	0.563	0.606
∑n-3	7.013	7.76	9.028	0.86	0.268	0.307	0.205
∑n-6	6.22	5.27	5.653	0.76	0.696	0.730	0.442
n-6:n-3	0.89	0.68	0.69	0.09	0.195	0.921	0.074
UFA:SFA	0.70	0.91	0.77	0.11	0.411	0.317	0.317
PUFA:SFA	0.23	0.25	0.26	0.03	0.790	0.782	0.535
Desaturase and elongase indices							
ID16	11.42	11.28	10.12	0.87	0.711	0.418	0.111
ID18	43.35	42.88	51.85	2.99	0.612	0.234	0.516
Elongation Index (EI)	60.13	59.74	65.29	3.03	0.875	0.324	0.198

^a,b,c^means having different superscript along the a row are significantly different (*P* < 0.05). ∑SFA = (C14:0 + C16:0 + C18:0), ∑MUFA = (C16:1+ C18:1+ C18:1 *trans*-11), ∑UFA = (C16:1+ C18:1+ C18:1 *trans*-11+ CLA *cis*-9 *trans*-11+ CLA *cis*-12 *trans*-10 + ∑n-3 + ∑n-6), ∑PUFA = (C18:1 *trans*-11+ CLA *cis*-9 *trans*-11+ CLA *cis*-12 *trans*-10 + ∑n-3 + ∑n-6), ∑n-3 = (C18:3n-3 + C20:5n-3 + C22:5n-3 + C22:6n-3), ∑n-6 = (C18:2n-6 + C20:4n-6) n-6:n-3 = (C18:2n-6 + C20:4n-6) ÷ (C18:3n-3 + C20:5n-3 + C22:5n-3 + C22:6n-3), UFA:SFA = (∑UFA)/∑SFA), PUFA:SFA = (∑PUFA/∑SFA). ID16, (Δ 9 desaturase enzyme activity on C16:0) = 100(C16:1)/(C16:0 + C16:1), ID18, (Δ 9 desaturase enzyme activity on C18:0) = 100(C18:1)/C18:0 + C18:1). EI = 100[(C18:0 + C18:1 n-9)/(C18:0 + C18:1n-9 + C16:0 + C16:1)] SEM = standard error of means. Values are least square means for 8 goats per diet. ^d^blend of 80 % canola oil and 20 % palm oil

**Table 11:** Least square means for the fatty acid composition (% of total FA) of subcutaneous fat in goats fed graded levels of blend of 80 % canola oil and 20 % palm oil

	Level of BCPO^a^ (%)				*P* value		
Parameter	0	4	8	SEM	Overall	Linear	Quadratic
C14:0, myristic	5.64	4.96	4.32	0.34	0.379	0.239	0.461
C16:0, palmitic	25.52	20.09	20.03	2.68	0.114	0.119	0.988
C16:1n-7, palmitoleic	3.57	3.20	2.86	0.24	0.126	0.073	0.327
C18:0, stearic	22.07	26.39	26.51	2.24	0.300	0.125	0.968
C18:1n-9, oleic	24.15	25.28	23.34	1.44	0.640	0.929	0.352
C18:1 *trans*-11 Vaccenic	3.15	3.19	3.45	0.56	0.833	0.713	0.637
CLA *cis*-9 *trans*-11	1.25	1.56	1.98	0.31	0.282	0.192	0.360
CLA *trans*-10 *cis*-12	1.88	2.03	1.74	0.26	0.739	0.970	0.443
C18:2n-6, linoleic	3.05	3.47	3.78	0.58	0.663	0.418	0.697
C18:3n-3, linolenic	2.04	1.60	1.63	0.38	0.679	0.386	0.955
C20:4n-6, arachidonic	2.60	1.89	2.32	0.43	0.512	0.356	0.492
C20:5n-3, eicosapentaenoic	2.80	2.20	3.16	0.68	0.607	0.890	0.328
C22:5n-3, docosapentaenoic	1.63	2.04	2.58	0.29	0.098	0.075	0.209
C22:6n-3, docosahexaenoic	1.78	2.71	2.01	0.48	0.371	0.329	0.311
Total FA (mg/g of fat)	526.53	579.41	549.34	87.20	0.428	0.425	0.320
FA sums and ratios							
∑SFA	53.23	51.43	50.86	2.11	0.944	0.741	0.956
∑MUFA	30.87	31.66	29.47	1.59	0.643	0.878	0.357
∑PUFA	17.02	17.49	19.20	2.06	0.612	0.325	0.667
∑n-3	8.24	8.54	9.89	0.80	0.679	0.833	0.395
∑n-6	5.65	5.36	6.37	1.18	0.810	0.514	0.446
n-6:n-3	0.74	0.66	0.67	0.04	0.911	0.530	0.916
UFA:SFA	0.83	0.86	0.87	0.08	0.658	0.677	0.919
PUFA:SFA	0.32	0.34	0.37	0.03	0.553	0.581	0.458
Desaturase and elongase indices							
ID16	12.27	13.74	12.49	0.98	0.291	0.348	0.237
ID18	52.25	48.92	46.82	2.12	0.100	0.222	0.277
Elongation Index (EI)	61.37	68.93	68.53	3.47	0.138	0.091	0.450

∑SFA = (C14:0 + C16:0 + C18:0), ∑MUFA = (C16:1+ C18:1+ C18:1 *trans*-11), ∑UFA = (C16:1+ C18:1+ C18:1 *trans*-11+ CLA *cis*-9 *trans*-11+ CLA *cis*-12 *trans*-10 + ∑n-3 + ∑n-6), ∑PUFA = (C18:1 *trans*-11+ CLA *cis*-9 *trans*-11+ CLA *cis*-12 *trans*-10 + ∑n-3 + ∑n-6), ∑n-3 = (C18:3n-3 + C20:5n-3 + C22:5n-3 + C22:6n-3), ∑n-6 = (C18:2n-6 + C20:4n-6) n-6:n-3 = (C18:2n-6 + C20:4n-6) ÷ (C18:3n-3 + C20:5n-3 + C22:5n-3 + C22:6n-3), UFA:SFA = (∑UFA)/∑SFA), PUFA:SFA = (∑PUFA/∑SFA). ID16, (Δ 9 desaturase enzyme activity on C16:0) = 100(C16:1)/(C16:0 + C16:1), ID18, (Δ 9 desaturase enzyme activity on C18:0) = 100(C18:1)/C18:0 + C18:1). EI = 100[(C18:0 + C18:1 n-9)/(C18:0 + C18:1n-9 + C16:0 + C16:1)]. SEM = standard error of means. Values are least square means for 8 goats per diet. ^a^blend of 80 % canola oil and 20 % palm oil

In the omental depot, the proportion of C14:0, C16:1n-7 and C18:1 *trans*-11 decreased (*P <* 0.05) as the level of BCPO increased in diet. Goats fed 4 and 8 % BCPO had similar (*P >* 0.05) proportion of *cis-*9 *trans*-11 CLA which was greater (*P <* 0.05) than those fed the control diet. The proportion of C18:3n-3 was greater (*P <* 0.05) in goats fed 4 % BCPO compared with those fed 0 and 8 % BCPO. The proportion of C20:5n-3 increased (*P <* 0.05) while the n-6/n-3 decreased (*P <* 0.05) with increasing level of BCPO in diet. Proportions of C16:0, C18:0, C18:2n-6, C20:4n-6, *trans-*10 *cis*-12 CLA, C22:5n-3 and C22:6n-3, total FA, sums and ratios of FA and the estimated elongase and desaturase indices were not affected (*P >* 0.05) by diet.

In the perirenal depot, the proportion of C14:0, C16:1n-7, C18:0 and total SFA decreased (*P <* 0.05) as the level of BCPO increased in diet. The proportion of C18:1n-9 and CLA *cis-*9 *trans*-11, total MUFA and UFA:SFA increased (*P <* 0.05) with increasing level of BCPO in diet. The control goats had higher (*P <* 0.05) ID 16 compared to those fed 4 and 8 % BCPO. The ID 18 increased linearly (*P <* 0.05) as the level of BCPO increased in diet. No significant (*P >* 0.05) changes were observed for total FA, proportion of C16:0, C18:2n-6, C18:3n-3, C20:4n-6, C22:5n-3, C22:6n-3 and *trans-*10 *cis*-12 CLA and the estimated elongase activity across the treatments.

In the mesentery depot, the proportion of C14:0 decreased (*P* < 0.05) as the level of BCPO increased in diet. The proportion of C18:1n-9 in goats fed 8 % BCPO was significantly higher (*P <* 0.05) compared with those fed 0 and 4 % BCPO. Goats fed 4 and 8 % BCPO had greater (*P <* 0.05) proportion of C20:5n-3 than the control goats. No significant differences (*P >* 0.05) were observed between diets with respect to the proportions of C16:0, C16:1n-7, C18:0, C18:2n-6, C20:4n-6, C18:3n-3, C22:5n-3, C22:6n-3, C18:1 *trans*-11, *cis-*9 *trans*-11 CLA and *trans-*10 *cis*-12 CLA and total FA. Similarly, estimated elongase and desaturase indices were not affected (*P* > 0.05) by diet.

In the subcutaneous depot, dietary BCPO was not a significant source of variation (*P* > 0.05) influencing the proportions of all FA, sums and ratios of FA and the estimated elongase and desaturase indices. However, the proportions of C14:0 and C16:1n-7 tended (*P* = 0.07) to decrease while that of C22:5n-3 tended (*P* = 0.07) to increase with increase in the level of BCPO in diet.

## Discussion

### Carcass traits, meat yield and adipose tissue partitioning

Dietary BCPO had no effect on live, empty and carcass weights, carcass linear measurements, and meat yield in goats. This finding could be due to the similarity in the slaughter weight across dietary treatments. Slaughter weight is one of the major determinants of carcass weight and meat yield in ruminants [23, 37]. The similarity in slaughter weight was likely due to the similar nutrient composition of diets fed to the goats. This finding is consistent with the similarity in the feed efficiency and average daily gain observed in a companion feeding trial [18]. As found in the current study, dietary sunflower oil (2.5 %) compared with the control diet did not affect the dressing percentage and carcass measurements in goats [3]. Similarly, Bock et al. [10] observed that 3.5 % tallow or soybean soap stock versus control diet did not affect dressing percentage in steers. Also, goats fed 4.5 % soybean oil or sunflower oil had similar carcass traits as those fed control diet [35]. In contrast, decreased dressing percentage and hot carcass weight were observed in steers fed 4 % soybean oil [40] and 4 and 8 % animal-vegetable oil blends [39] while carcass yield increased in steers fed 3.5 % soybean oil, tallow or yellow grease versus steers fed control diet [2] and Nellore steers fed soybean and linseed oils or their calcium salts compared to those fed control diet [1]. The dressing percentage (based on empty body weight) of the goats ranged from 53.55 to 54.56 %. These values are similar to those reported by Marinova et al. [3] and Kadim et al. [23].

The similarity in the back fat thickness, intramuscular fat and the weights of adipose tissues across dietary treatments suggests that dietary supplementation of BCPO did not alter the deposition and distribution of fat in the adipose tissues of goats. This finding could be attributed to the similar metabolizable energy content of the diets. Feed energy supplied in excess of the basal requirement can increase fat deposition in animals [41]. The major substrate for *de novo* fatty acid synthesis in tissues is glucose [42]. Thus, diets capable of promoting glucose supply to the tissues might increase fat deposition. Propionate is a gluconeogenic precursor and thus could increase supply of glucose to the tissues [42, 43]. Erstwhile companion studies have shown that supplementation of BCPO did not affect *in vitro* [17] and *in vivo* [18] ruminal molar concentration of propionate. These observations lend credence to the similar fat deposition across the treatments. The current observation contrasts past studies [2, 39] wherein oil supplementation increased back fat thickness and weight of adipose tissues. In line with the current findings, dietary palm oil or its calcium soap compared with the basal diet did not affect the weights of omental and perirenal fat in lambs [37]. Marinova et al. [3] observed that dietary supplementation of 2.5 % sunflower oil did not affect the weight of perirenal fat, sweetbread and caul but increased intramuscular fat in lambs. The discrepancies between the current findings and the earlier reports could be due to differences in the amounts and fatty acid composition of the dietary lipids used.

The similarity in the weights of adipose tissues across dietary treatments corroborates the similarity in the moisture content of adipose tissues which suggests that supplementation of BCPO in diet did not play a significant role in the maturation of adipose cells. High proportion of water in adipose tissues signifies a less mature tissue [41]. Bas et al. [6] observed that the moisture content of omental and perirenal adipose tissue of goats raised indoors with concentrate (high fat diet) was lower than those raised outdoors with concentrate and grass or with only Argan pulp (low fat diet) suggesting that high fat diets may increase hyperplasia and hypertrophy of adipose cells.

Dietary BCPO had no effect on color coordinates especially yellowness and the concentration of total carotenoid in adipose tissues in goats. This observation is unexpected given the increase in dietary fat and carotenoid contents of the diet as the level of BCPO increased, and the 100 d feeding duration employed in this study. Diet and feeding duration are some of the important factors influencing the yellowness of adipose tissues in ruminants [44]. The absorption and deposition of carotenoid in various tissues in ferrets was positively correlated with dietary fat [45]. However, mammals differ in their ability to absorb carotenoids [46]. For example, humans can accumulate carotenes and xanthophylls in their adipose tissues without discrimination, cattle and horses can accumulate only carotenes while goats and sheep accumulate neither carotenes nor xanthophylls [46]. In addition, Mora et al. [47] reported that goats have higher levels of jejunal and duodenal 15, 15’dioxygenase activities than cattle when fed dietary beta-carotene which may explain the lower pigmentation of adipose tissue in goats compared with cattle. The 15, 15’dioxygenase is the enzyme responsible for the conversion of beta-carotene to retinal [47].

### Tissue fatty acid composition

The management strategies employed in the current study were successful in ensuring similar carcass weight, carcass fatness and total fatty acids in all the tissues examined. Thus, the observed FA composition in the examined tissues was not confounded by carcass fatness.

Regardless of the diet, the most abundant FA in SS was C18:1n-9 and its concentration was influenced by diet. This observation is in tandem with previous studies in goats [35], sheep [36] and cattle [38] in which C18:1n-9 was identified as the most abundant FA in muscle following dietary oil supplementation. The increase in C18:1n-9 in response to BCPO could be due to the increase in dietary C18:1n-9 and/or increase in the Δ 9 desaturase enzyme activities (ID 18) necessary for the de novo conversion of C18:0 to C18:1n-9. This observation is consistent with those observed in a companion *in vitro study* where the ruminal concentration of C18:1n-9 after 24 h incubation increased as the level of BCPO increased in the substrate [17]. As found in the current study, supplementation of 3.3 % canola oil, canolamide or canola oil-canolamide mix oil increased the concentration of C18:1n-9 in bovine milk [5]. Conversely, 3 % dietary canola oil compared with 3 % palm oil did not affect the concentration of C18:1n-9 in *longissimus lumborum* muscle from goats [48].

Dietary supplementation of BCPO depressed the concentration of C16:0 and C16:1n-7 in SS muscle. These observations concur with those of Loor et al. [5] and Ferlay et al. [49]. Depression of medium chain FA in response to dietary unsaturated fats could be due to the inhibition of the activities of lipogenic enzymes required for the synthesis of medium chain FA or the preferential incorporation of long chain FA from diet and/or adipose tissues [4, 5, 49, 50]. This lends credence to the significant decrease in the Δ 9 desaturase activity (ID 16) which coincides with the linear decrease in the concentration of C16:0 and C16:1 as BCPO increased in diet. Palmitic acid (C16:0) can be converted to C16:1n-7 by Δ 9 desaturase [51].

The concentration of C18:1 *trans*-11 Vaccenic and CLA cis-9 *trans*-11 in SS increased as BCPO increased in diet. This observation could be due to the increase in the dietary concentration of unsaturated fatty acids. Both C18:1 *trans*-11 Vaccenic and CLA *cis*-9 *trans*-11 are intermediate products of ruminal biohydrogenation of unsaturated FA [4, 13, 14]. Feeding unsaturated FA causes incomplete biohydrogenation of unsaturated FA accompanied with biohydrogenation intermediates [13, 14]. In addition, it has been established that Δ 9 desaturation of C18:1 *trans*-11 Vaccenic in adipose tissue could yield CLA cis-9 *trans*-11 [4, 16, 17]. In line with the current observation, supplementation of 3.3 % canola oil, canolamide or canola oil-canolamide mix (Loor et al. [5] and 1 kg/day canola oil [50] increased the concentration of C18:1 *trans*-11 Vaccenic and CLA *cis*-9 *trans*-11 in bovine milk compared with the control diet.

The proportion of C18:0, C18:2n-6 and C20:4n-6 in SS was not influenced by diet. In contrast, the concentration of C18:3n-3 and C20:5n-3 increased linearly in response to dietary BCPO. Similarly, Karami et al. [48] found that 3 % canola oil compared with 3 % palm oil did not affect the concentration of C18:0, C18:2n-6 and C20:4n-6 but enhanced the concentration C18:3n-3 in *longissimus lumborum* muscle from goats. However, the authors did not observe increment in the concentration of long chain n-3 FA. The linear increase in the proportion of C18:3n-3 in goats fed 4 and 8 % BCPO relative to those fed control diet could be responsible for the increase in the proportion of C20:5n-3 in their muscles since the FA is a metabolite of C18:3n-3 [4] suggesting *in vivo* elongation and desaturation. This coincides with the linear increase in the elongation index in response to supplementation of BCPO. This observation is consistent with those of Gjerlaug-Enger et al. [52] who observed that dietary rapeseed products enhanced the concentration of C18:3n-3, C20:5n-3 and C22:5n-3 in *longissimus dorsi* muscle of pork compared with the control diet.

The linear increase in total n-3 PUFA was accompanied by a linear decrease in n6/n3 ratio as the level of BCPO increased in the diet. The n6/n3 ratio for the 4 and 8 % BCPO was within the range of the recommended value (<4) for healthy diet [53]. Dietary BCPO enhanced the total MUFA and decreased the total SFA in SS muscle. Diet had no effect on the total PUFA and total FA. The PUFA/SFA ratio increased with increase in dietary BCPO. This could be attributed to the decrease in the SFA in BCPO based diets. Regardless of diet, the PUFA/SFA ratio of SS was within the range of the recommended value (>0.4) [53].

The major fatty acids in all adipose tissues were C16:0, C18:0 and C18:1n-9. This observation is in line with those of Bas et al. [6] and Castro et al. [37]. The decrease in the proportion of C14:0 and C16:1n-7 in the perirenal and omental fats from goats fed 4 and 8 % BCPO relative to the control goats could be due to the reduction in the mRNA abundance and activity of lipogenic enzymes required for synthesis of medium chain FA [4]. The decrease in the proportion of C16:1n-7 coincides with the linear decrease in the Δ 9 desaturase activity (ID 16) as BCPO increased in the diets. Dietary BCPO deceased the proportion of C18:0 and total SFA and enhanced the concentration of C18:1n-9, CLA cis-9 *trans*-11 and total MUFA in perirenal fat. In omental fat, dietary BCPO increased the concentration of CLA cis-9 *trans*-11, C20:5n-3 and total PUFA. In the mesentery fat, goats fed 8 % BCPO had higher C18:1n-9 and Δ 9 desaturase activity (ID 18) compared to other diets. Also, BCPO caused a linear increase in the proportion of C20:5n-3. The current observation is consistent with those of Stanford et al. [54] who observed that increasing the level of canola screening in diets reduced the concentration of C14:0, C16:0, C16:1n-7, C18:0 and total SFA and enhanced that of C18:1n-9, C18:3n-3, total MUFA and PUFA in perirenal adipose tissue in lambs. In contrast, dietary rapeseed oil, linseed oil or hydrogenated rapeseed oil did not alter the fatty acid composition of perirenal adipose tissues in lambs [55].

Contrary to the findings in perirenal, mesentery and omental adipose tissues, dietary BCPO did not affect the FA composition of subcutaneous adipose tissue. This finding concurs with those of Potkański et al. [55] who observed that dietary rapeseed oil did not alter the FA composition of subcutaneous adipose tissues in lambs. Similarly, dietary fish oil did not alter the FA composition of subcutaneous adipose tissue in lambs [56]. Contrary to the present findings, earlier trials have reported significant alterations in the FA composition of subcutaneous adipose tissue when oils high in unsaturated FA were fed to sheep [57] and goats [35]. The inconsistencies observed in the FA composition of adipose tissues in response to dietary BCPO reflect variation in FA metabolism in the adipose tissues.

## Conclusions

The results of this study showed that dietary supplementation of blend of 80 % canola oil and 20 % palm oil in diet did not affect carcass traits, fat color, and fat deposition and distribution in goats. Thus, dietary BCPO is not an effective repartitioning agent for fat in goats. However, BCPO altered the fatty acid composition of *supraspinatus* muscle, mesentery, omental and perirenal adipose tissues in goats.

## Abbreviations

BCPO: Blend of 80 % canola oil and 20 % palm oil
FA: Fatty acid
SS: Supraspinatus muscle
SFA: Saturated fatty acid
MUFA: Monounsaturated fatty acids
PUFA: Polyunsaturated fatty acids
